# The Effect of Autologous Dendritic Cell Therapy on Renal Perfusion in Diabetic Kidney Disease: Analysis of Doppler Ultrasound and Angiogenesis Biomarkers

**DOI:** 10.3390/diseases13040116

**Published:** 2025-04-16

**Authors:** Ardianto Pramono, Jonny, Djuwita Adi Wahyono, Aditya Pratama Lokeswara, Enda Cindylosa Sitepu, Ermi Girsang, Terawan Agus Putranto

**Affiliations:** 1 Faculty of Medicine, Dentistry, and Health Sciences, Universitas Prima Indonesia, Medan 20118, Indonesia; ardiantorspad@gmail.com (A.P.); jonny@unprimdn.ac.id (J.); 2 Faculty of Medicine, National Development University “Veteran” Jakarta, Jakarta 12450, Indonesia; djuwitaadiw@gmail.com; 3 Department of Radiology, Gatot Soebroto Central Army Hospital, Jakarta 10410, Indonesia; lokeswaraaditya@gmail.com; 4 Faculty of Military Medicine, University of Defense, Bogor 16810, Indonesia; 5 Division of Nephrology, Department of Internal Medicine, Gatot Soebroto Army Hospital, Jakarta 10410, Indonesia; 6 Indonesia Army Cellcure Center, RSPAD Gatot Soebroto Jakarta, Jakarta 10410, Indonesia; endacsitepu@gmail.com

**Keywords:** diabetic kidney disease, dendritic cells, pulsative index, resistive index, vascular endothelial growth factor, endothelin

## Abstract

Background: Diabetes mellitus (DM) is a global health challenge with a severe health burden. Approximately 40% of diabetic patients develop diabetic kidney disease (DKD), leading to kidney failure. Autologous dendritic cell therapy may enhance renal function by modulating vascular markers. Methods: Involving 35 patients, this quasi-experimental study assessed the pulsatility index (PI), resistive index (RI), vascular endothelial growth factor (VEGF), and endothelin levels before and four weeks following autologous dendritic cell administration. Results: A significant reduction in median PI was found from 1.61 ± 0.63 to 1.21 ± 0.26 (*p* < 0.001). The increase in mean RI was insignificant from 0.74 ± 0.07 to 0.75 ± 0.06 (*p* = 0.17). The median VEGF showed a slight reduction from 522.10 ± 608.6 to 473.70 ± 550 (*p* = 0.589) and endothelin from 1.74 ± 0.71 to 1.63 ± 0.76 (*p* = 0.554). Conclusions: This study shows that autologous dendritic cell therapy may improve kidney perfusion in DKD patients, indicated by a significant reduction in the PI. These findings suggest potential therapeutic benefits for renal perfusion in DKD.

## 1. Introduction

Type 2 diabetes is a complex chronic disease caused by insulin resistance or insufficient insulin production, closely associated with lifestyle and environmental factors. It has become one of the fastest-growing global health concerns [1]. The prevalence of DM type 2 in Indonesia has reached 10.8% in 2021, positioning the country within the top 10 for the highest diabetes prevalence worldwide. It is estimated that approximately 463 million adults aged 20–79 years suffer from DM type 2, while the number is projected to rise to 578 million by 2030 [2]. Previous studies indicate that around 40% of type 2 diabetes patients develop complications, such as DKD, with over half suffering from moderate-to-severe (stages 3 and 4) DKD [3].

DKD is typically caused by microvascular damage related to diabetes, and it is one of the leading causes of kidney failure globally. DKD is typically defined by persistent albuminuria (urinary albumin excretion of more than 30 mg/24 h or a urine albumin-to-creatinine ratio exceeding 30 mg/g), a sustained reduction in the estimated glomerular filtration rate (eGFR) less than 60 mL/min per 1.73 m^2^, or a combination of both for at least three months [3]. The pathophysiology of DKD is highly complex, involving metabolic, hemodynamic, inflammatory, and fibrotic pathways that collectively drive oxidative stress and compromise renal perfusion. The hemodynamic component, which is mediated by the renin–angiotensin–aldosterone system (RAAS), angiotensin II, endothelin-1, and the buildup of advanced glycation end-products (AGEs), is a crucial part of this process. These factors collectively act to worsen renal hypoperfusion, further complicating the disease’s progression [4].

Radiological modalities, such as Doppler ultrasound, can be utilized to assess renal perfusion. The essential parameters for evaluating intrarenal hemodynamics are the RI and PI [5]. Measuring the RI and PI of the renal interlobar arteries is essential in assessing intrarenal perfusion in DKD, as they can describe intrarenal vasoconstriction that may predict complications in the future [6].

Furthermore, previous studies have suggested that angiogenesis markers, such as vascular endothelial growth factor (VEGF) and endothelin, are emerging indicators for predicting the progression of diabetic nephropathy due to their close association with renal perfusion [7]. Endothelin is essential in vasoconstriction, inflammation, and kidney fibrosis, which can negatively impact glomerular filtration and increase proteinuria levels. Meanwhile, VEGF is essential in maintaining renal perfusion in both standard and pathological conditions. It is crucial in angiogenesis to sustain the microvascular structure of the kidney [8,9,10,11].

Our previous study revealed that administering autologous dendritic cells can significantly reduce UACR in DKD patients. Decreases in TNF-α, a potent driver of inflammation, were also found after the administration of autologous dendritic cells, which strongly suggests the cells can induce inflammation controls that eventually lead to an improvement in UACR [12]. However, the analysis of endothelial biomarkers (ICAM, VCAM, and VEGF) revealed differential responses in specific subgroups, mainly based on the classification of albuminuria and glycemic status [13]. Furthermore, water molecule diffusion was measured using Magnetic Resonance Imaging (MRI) to assess whether the treatment induced structural changes in the kidney. However, no significant changes were detected [14]. This finding suggests that the reduction in albuminuria following autologous dendritic cell administration was not attributable to improvements in kidney structure as indicated by water molecule diffusion. Their impact on renal perfusion and angiogenesis markers needs to be examined to further elucidate the mechanism of action underlying the therapeutic effect of autologous dendritic cells. Hence, this study aims to analyze the differences in renal perfusion in DKD patients by using ultrasound to assess changes in RI and PI, as well as differences in angiogenesis markers, including VEGF and endothelin, before and after autologous dendritic cell administration.

## 2. Materials and Methods

### 2.1. Study Design

This study constitutes experimental research utilizing a quasi-experimental design, specifically a one-group pre-test post-test design, in which no randomization or blinding was applied. The procedures were conducted according to applicable regulations. The protocols were approved by the Ethics Committee of the Indonesia Central Army Hospital Gatot Soebroto (RSPAD) with ethical clearance letter number 101/VIII/KEPK/2024. This study is registered at ClinicalTrials.gov with the identifier NCT06866158. All subjects provided written informed consent before participation.

### 2.2. Study Subjects

The study sample comprised all DKD patients at RSPAD who qualified for the inclusion criteria. These criteria included a diagnosis of type 2 diabetes mellitus (DM) based on Indonesian guidelines, an eGFR of ≥30 mL/min/1.73 m^2^, and a urinary albumin-to-creatinine ratio (UACR) of ≥30 mg/g for the proteinuria group. The exclusion criteria were patients undergoing immunosuppressive therapy, those with other kidney diseases or conditions causing proteinuria, patients with different types of DM, positive pregnancy tests, individuals with immunodeficiency disorders, those with invasive cancer receiving non-hormonal treatment, patients with a history of thromboembolism, or those with physical or mental disabilities limiting daily activities, as well as any medical condition that would hinder participation.

### 2.3. Study Protocol

Each subject participated in a five-week clinical trial, starting with the screening phase, laboratory examinations, Doppler ultrasound assessments, blood collection for autologous dendritic cell preparation, and autologous dendritic cell injections. Laboratory assessments and Doppler ultrasound evaluations were conducted four weeks after the autologous dendritic cell injection.

### 2.4. Laboratory Examination

Angiogenesis biomarkers that affect renal perfusion, namely VEGF and endothelin, were measured using a sandwich ELISA (Reed Biotech Ltd., Wuhan, China) from serum blood samples collected before and four weeks after the autologous dendritic cell injection.

### 2.5. Doppler Ultrasound Examination

**Figure 1:**
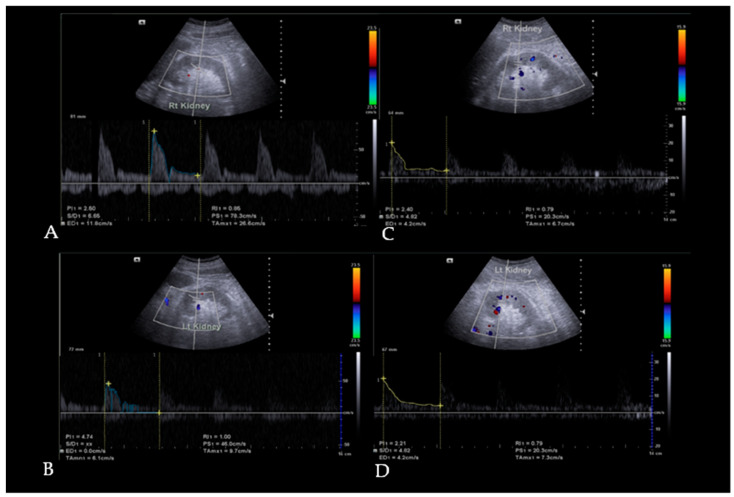
This picture illustrates Doppler ultrasound findings demonstrating changes in the resistive index (RI) and pulsatility index (PI) in diabetic kidney disease (DKD) patients following autologous dendritic cell administration. The Doppler ultrasound was performed on the interlobar arteries of both kidneys to assess RI and PI. Pre-treatment (**A**,**B**), the right kidney exhibited an RI of 0.86 and a PI of 2.50, while the left had an RI of 1.00 and a PI of 4.74. Post-treatment (**C**,**D**), RI and PI decreased bilaterally, with the right kidney showing an RI of 0.79 and a PI of 2.40 and the left kidney demonstrating an RI of 0.79 and a PI of 2.21.

Doppler ultrasound assessments were performed supine, with Siemens Acuson NX3^®^ ultrasound utilizing a curvilinear probe in spectral Doppler mode, with an insonation angle of less than 60° between the transducer and blood flow. These assessments measured the RI and PI of the right and left interlobar renal arteries. Images obtained from the examinations are illustrated in Figure 1. Two radiologists conducted the examinations before the autologous dendritic cell injection and four weeks afterward.

### 2.6. Dendritic Cells (DC) Preparation

A total of 40 cc of peripheral blood was collected from each subject, from which monocytes were isolated using Lymphoprep^TM^ (StemcellTM Technologies Inc., Vancouver, BC, Canada). Granulocyte Macrophage Colony Stimulating Factor and Interleukin-4 (Aivita Biomedical, Irvine, CA, USA) were added to the culture medium for five days at 37 °C with 5% CO_2_. Then, antigens were added for two days to initiate maturation (Aivita Biomedical, Ievine, CA, USA). We did not separate monocytes and lymphocytes during the culture process to allow lymphocyte activation. As a result, the final cell product consisted of a mixed population of dendritic cells and lymphocytes. The flow cytometry (FACS) results demonstrating the cell product specification are provided in Supplementary Figure S1.

### 2.7. Statistical Analysis

Data analysis was conducted using either an independent *t*-test or a Wilcoxon test, which was selected based on the outcome of the normality test. All statistical analyses were performed using the Statistical Packages for Social Science (SPSS^®^) software version 25.

## 3. Results

### 3.1. Subject Characteristic

The study involved 35 participants, of which 22 were female (62.9%) and 13 were male (37.1%). The majority of the participants were over the age of 60, with 23 individuals in this category, while 12 were under the age of 60. The median body weight was 65 kg, and the median height was 158 cm. The participants had normal albuminuria, with 21 (60.0%) presenting with microalbuminuria and 14 (40.0%) with macroalbuminuria. The severity of CKD was also assessed, with 16 participants (45.7%) in CKD stages 1 and 2, while 19 participants (54.3%) were in CKD stage 3 (Table 1).

### 3.2. Resistive Index (RI) and Pulsatility Index (PI) Changes

This study assessed renal perfusion using Doppler ultrasound, measuring the RI and PI at two points, before and four weeks after autologous dendritic cell administration. A comprehensive Doppler ultrasound evaluation was performed for each subject to ensure measurement consistency.

The overall mean ± standard deviation (SD) of RI before treatment was 0.74 ± 0.07, which increased to 0.75 ± 0.06 four weeks post-treatment, though this change was not statistically significant (*p* = 0.17). Subgroup analysis based on urinary albumin-to-creatinine ratio (UACR) showed that in the microalbuminuria group, the mean ± SD RI was 0.74 ± 0.07 before treatment and 0.75 ± 0.07 after treatment (*p* = 0.215). In the macroalbuminuria group, the pre-treatment RI was 0.74 ± 0.07, increasing to 0.76 ± 0.04 post-treatment (*p* = 0.465).

Further stratification by chronic kidney disease (CKD) stage revealed that in patients with CKD stages 1 and 2, the mean ± SD RI was 0.73 ± 0.05 pre-treatment and 0.74 ± 0.05 post-treatment (*p* = 0.295). Among those with CKD stage 3, the RI increased from 0.75 ± 0.08 pre-treatment to 0.76 ± 0.06 post-treatment (*p* = 0.327). These results indicate that while a slight increase in RI was observed across all groups, the changes were not statistically significant (Table 2).

Doppler ultrasound assessed the PI before and four weeks after autologous dendritic cell administration. Overall, the pre-treatment PI had a median of 1.61 ± 0.63, significantly decreasing to 1.21 ± 0.26 post-treatment (*p* < 0.001).

Subgroup analysis based on urinary albumin-to-creatinine ratio (UACR) categories showed that in the microalbuminuria group, the median PI decreased from 1.57 ± 0.49 pre-treatment to 1.22 ± 0.50 post-treatment, with a statistically significant *p*-value of <0.001. In the macroalbuminuria group, the mean ± standard deviation (SD) PI was 1.83 ± 0.67 pre-treatment, significantly decreasing to 1.23 ± 0.14 post-treatment (*p* = 0.004).

**Figure 2:**
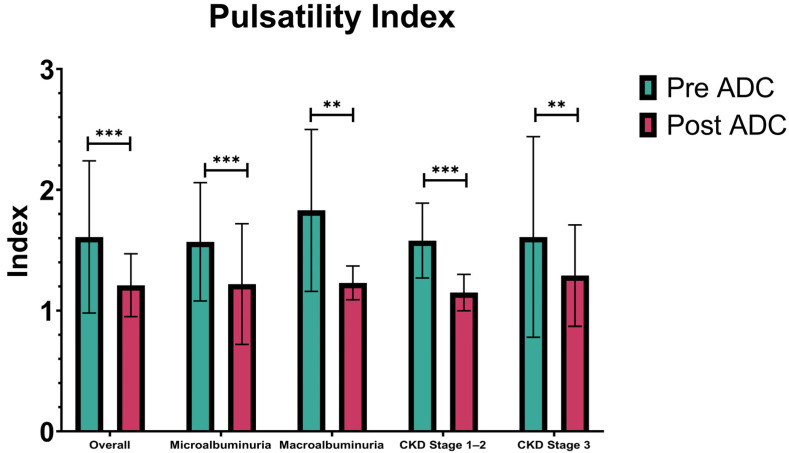
Pulsatility index. Data from overall, microalbuminuria, and CKD stage 3 are presented as the median ± IQR, and *p*-values were calculated by Wilcoxon signed rank. Meanwhile, data from macroalbuminuria and CKD Stages 1–2 are presented as the mean ± standard deviation, and the *p*-value was calculated by paired *t*-test. ** *p* < 0.01, *** *p* < 0.001. ADC: autologous dendritic cell, CKD: chronic kidney disease.

Stratification based on CKD stages revealed that in CKD stages 1 and 2, the mean ± SD PI decreased from 1.58 ± 0.31 pre-treatment to 1.15 ± 0.15 post-treatment (*p* < 0.001). Among patients with CKD stage 3, the median PI was 1.61 ± 0.83 pre-treatment, significantly decreasing to 1.29 ± 0.423 post-treatment (*p* = 0.002). These findings indicate a significant reduction in PI across all subgroups following autologous dendritic cell therapy (Figure 2).

### 3.3. Angiogenesis Biomarker Changes

Angiogenesis biomarkers, including vascular endothelial growth factor (VEGF) and endothelin, were measured in this study. Each subject underwent comprehensive laboratory testing for VEGF and endothelin at two points before and four weeks after autologous dendritic cell administration.

VEGF levels were analyzed before and after treatment. In the overall analysis, the median VEGF level was 522.10 ± 608.6 pg/mL pre-treatment, slightly decreasing to 473.70 ± 550 pg/mL post-treatment. However, this change was not statistically significant (*p* = 0.589).

Subgroup analysis based on the urinary albumin-to-creatinine ratio (UACR) showed that in the microalbuminuria group, the pre-treatment VEGF median was 305.05 pg/mL, increasing slightly to 318.10 pg/mL post-treatment (*p* = 0.913). In the macroalbuminuria group, the mean ± standard deviation (SD) VEGF level was 611.72 ± 319.10 pg/mL pre-treatment, decreasing marginally to 604.35 ± 295.19 pg/mL post-treatment, with no significant difference (*p* = 0.863).

When stratified by CKD stage, patients with CKD stages 1 and 2 had a mean ± SD VEGF level of 454.44 ± 313.36 pg/mL pre-treatment and 456.71 ± 290.65 pg/mL post-treatment (*p* = 0.94). In CKD stage 3, the mean VEGF level was 618.35 ± 376.17 pg/mL before treatment and 592.22 ± 316.27 pg/mL after treatment, with no statistically significant difference (*p* = 0.498). These findings indicate that VEGF levels remained unchanged following autologous dendritic cell therapy across all subgroups (Table 3).

Our previous study has revealed that administering autologous dendritic cells can significantly reduce UACR in DKD patients. Decreases in TNF-α, a potent driver of inflammation, were also found after administration of autologous dendritic cells, which strongly suggests the cells can induce inflammation controls that eventually lead to improvement of UACR [12].

Endothelin levels were measured before and four weeks after autologous dendritic cell administration. In the overall analysis, the median endothelin level was 1.74 ± 0.71 pg/mL pre-treatment, slightly decreasing to 1.63 ± 0.76 pg/mL post-treatment. However, this change was not statistically significant (*p* = 0.554).

Subgroup analysis based on urinary albumin-to-creatinine ratio (UACR) categories showed that in the microalbuminuria group, the mean ± standard deviation (SD) endothelin level was 1.70 ± 0.47 pg/mL before treatment, decreasing slightly to 1.65 ± 0.60 pg/mL post-treatment (*p* = 0.570). In the macroalbuminuria group, the pre-treatment mean ± SD endothelin level was 1.91 ± 0.43 pg/mL, with a slight post-treatment decrease to 1.89 ± 0.47 pg/mL, which was not statistically significant (*p* = 0.847).

When stratified by CKD stage, patients with CKD stages 1 and 2 had a mean ± SD endothelin level of 1.62 ± 0.36 pg/mL pre-treatment, which slightly decreased to 1.59 ± 0.38 pg/mL post-treatment (*p* = 0.604). In CKD stage 3, the mean ± SD endothelin level was 1.93 ± 0.49 pg/mL before treatment, decreasing slightly to 1.89 ± 0.64 pg/mL post-treatment (*p* = 0.712). These results indicate that endothelin levels remained unchanged following autologous dendritic cell therapy across all subgroups (Table 4).

## 4. Discussion

In this study, we found that after autologous dendritic cell administration, the RI change was insignificant [from 0.74 ± 0.07 to 0.75 ± 0.06 (*p* = 0.17)]. RI is an indicator of renal resistance to perfusion and reflects arterial resistance. The normal range of RI values for the kidney is between 0.47 and 0.70, with the difference between the right and left kidneys not exceeding 5–8%. This value may increase with age or in renal disease conditions [15]. The resistive index reflects the differences between maximum and minimum blood flow velocities and the complex interaction between systemic circulation and renal microcirculation. An increase in RI indicates greater arterial stiffness and elevated pulsatility [15]. Based on several previous studies, changes in RI following revascularization occur within approximately 3 months [16].

However, administering autologous dendritic cells significantly reduced the median PI value from 1.61 ± 0.63 to 1.21 ± 0.26 (*p* < 0.001). This decrease indicates a significant change in blood flow dynamics or vascular resistance. Microangiopathy complications and renal dysfunction can be predicted by measuring the PI value with Doppler ultrasound. The decrease in PI observed in this study indicates a significant change in blood flow dynamics or vascular resistance, as an elevated PI value can predict kidney damage and increased vascular resistance associated with higher protein leakage into the urine. Likewise, lower PI values are generally associated with improved blood flow and reduced resistance within the vascular system [17]. The statistically significant decrease in PI following this therapy indicates that autologous dendritic cell has the potential as an adjunctive treatment to improve renal blood flow and reduce vascular resistance in patients with CKD [18]. This suggests that the albuminuria-lowering effect of autologous dendritic cell therapy was mediated by decreased vascular resistance in the kidney.

On the other hand, the administration of autologous dendritic cells had a similar effect on perfusion biomarkers VEGF and endothelin. The median VEGF value decreased from 522.10 ± 608.6 to 473.70 ± 550 (*p* = 0.589), and the mean endothelin value decreased from 1.79 ± 0.46 to 1.75 ± 0.55 (*p* = 0.554). However, these changes were not statistically significant. This is a positive impact of autologous dendritic cell administration on vascular regulation, potentially reducing the risk of vascular complications. The reduction in endothelin and VEGF suggests that autologous dendritic cells may have a beneficial modulatory effect on endothelial function. Previous studies have revealed that endothelin acts as a potent vasoconstrictor and is associated with increased blood pressure and cardiovascular risk [18]. Meanwhile, VEGF plays a significant role in the pathophysiology of DKD. In kidneys, VEGF-A is primarily expressed by podocytes and affects the structure and function of glomerular endothelial cells (GECs) by binding to its receptor, VEGFR-2. In early DKD, increased VEGF-A expression leads to glomerular damage, promoting proteinuria and endothelial dysfunction. While VEGF-A signaling can stimulate new blood vessel formation, its overactivation contributes to kidney damage, and the inhibition of VEGF in diabetic models has been shown to reduce proteinuria and mitigate glomerular injury [8]. Reducing endothelin levels may significantly impact renal function, particularly CKD. Endothelin-1 (ET-1) is a potent vasoconstrictor significantly affecting renal blood flow. Its overexpression can lead to decreased GFR, increased proteinuria, and podocyte damage. This primarily occurs through the activation of endothelin type A (ETA) receptors, which trigger vasoconstriction of afferent arterioles, hyperfiltration, and eventually expedited kidney damage [19,20]. Reducing endothelin levels may enhance GFR by decreasing vascular resistance in the kidneys [21,22]. However, despite a significant decrease in PI, non-significant changes in these markers suggest that other factors might mediate decreased vascular resistance.

The pathogenesis of DKD involves multiple factors, such as chronic inflammation, endothelial dysfunction, fibrosis, and changes in renal perfusion. Studies have found that the administration of autologous DC can decrease UACR [12]. In this study, we studied renal perfusion and angiogenesis parameters to elucidate the mechanism of action on how autologous DC can decrease UACR. Although administering autologous dendritic cells may affect other aspects of renal or circulatory health, its impact on RI, VEGF, and endothelin was not statistically significant. However, it should be considered that autologous dendritic cells may require a longer duration to influence the regulation of RI, VEGF, and endothelin or that the reparative mechanisms induced by these cells are more complex and do not directly affect RI, VEGF, and endothelin significantly within the context of this study. Changes in RI and PI may evolve over a more extended period—potentially up to 3 months or more—which may partially explain the non-significant findings observed in the present study, which was designed as a short-term exploratory analysis. Nonetheless, the significant change observed in PI in this cohort provides a promising signal. It supports the rationale for conducting future studies with more extended follow-up periods to better assess the sustained effects and clinical relevance of dendritic cell therapy in DKD. Additionally, further research with a larger sample size is needed to explore other factors that may influence the response to autologous dendritic cells and their impact on managing vascular-related clinical conditions and understand the long-term effects of this therapy on CKD patients.

## 5. Conclusions

This study evaluates the effects of autologous dendritic cell therapy on kidney perfusion in patients with DKD, focusing on changes in vascular markers. The results demonstrated a significant decrease in PI, indicating improved kidney perfusion. At the same time, changes in the RI, VEGF, and endothelin levels were not statistically significant. These findings suggest that the therapeutic mechanism of autologous dendritic cell therapy in DKD may involve the modulation of renal perfusion.

## Figures and Tables

**Table 1:** Subject characteristics. Gender, age group, body mass index classification, UACR classification, and CKD grade are given as *n* (%), while age, weight, and height are given as the median (min–max).

Characteristics	Category	Description (*n* = 35)
Gender	Female	22 (62.9%)
	Male	13 (37.1%)
Age group	<60	12 (34.2%)
	>60	23 (65.7%)
Age		62 (44–83) years
Weight		65 (41–101) kg
Height		158 (145–181) cm
Body mass index classification	Underweight	2 (5.7%)
	Normal weight	9 (25.7)
	Overweight	0 (0%)
	Obesity I	14 (40%)
	Obesity II	10 (28.6%)
UACR classification	Normal albuminuria	0 (0%)
	Microalbuminuria	21 (60%)
	Macroalbuminuria	14 (40%)
CKD grade	Stages 1 and 2	16 (45.7%)
	Stage 3	19 (54.3%)

**Table 2:** Resistive index (RI). Data are presented as median ± interquartile range. Wilcoxon signed rank calculated *p*-value.

Subject Group		Pre-Autologous Dendritic Cells	Post-Autologous Dendritic Cells	*p*-Value
Overall		0.74 ± 0.07	0.75 ± 0.06	0.17
UACR	Microalbuminuria	0.74 ± 0.07	0.75 ± 0.07	0.215
	Macroalbuminuria	0.74 ± 0.07	0.76 ± 0.04	0.465
Stage CKD	CKD stages 1 and 2	0.73 ± 0.05	0.74 ± 0.05	0.295
	CKD stage 3	0.75 ± 0.08	0.76 ± 0.06	0.327

**Table 3:** Serum VEGF Level. Data are presented as the median ± interquartile range. Wilcoxon signed rank was used to calculate the *p*-value.

Subject Group		VEGF Pre-Autologous Dendritic Cells (pg/mL)	VEGF Post-Autologous Dendritic Cells (pg/mL)	*p*-Value
Overall (median ± IQR)		522.10 ± 608.6	473.70 ± 550	0.589
UACR	Microalbuminuria (median ± IQR)	305.05 ± 634.82	318.10 ± 533.32	0.913
	Macroalbuminuria (mean ± SD)	611.72 ± 319.10	604.35 ± 295.19	0.863
Stage CKD	CKD stages 1 and 2 (mean ± SD)	454.44 ± 313.36	456.71 ± 290.65	0.94
	CKD stage 3 (mean ± SD)	618.35 ± 376.17	592.22 ± 316.27	0.498

**Table 4:** Serum endothelin level. Data are presented as the median ± interquartile range. Wilcoxon signed rank was used to calculate the *p*-value.

		Endothelin Pre-Autologous Dendritic Cells (pg/mL)	Endothelin Post Autologous Dendritic Cells (pg/mL)	*p*-Value
General (median ± IQR)		1.74 ± 0.71	1.63 ± 0.76	0.554
UACR	Microalbuminuria (mean ± SD)	1.70 ± 0.47	1.65 ± 0.60	0.57
	Macroalbuminuria (mean ± SD)	1.91 ± 0.43	1.89 ± 0.47	0.847
Stage CKD	CKD stages 1 and 2 (mean ± SD)	1.62 ± 0.36	1.59 ± 0.38	0.604
	CKD stage 3 (mean ± SD)	1.93 ± 0.49	1.89 ± 0.64	0.712

## Data Availability

The original contributions presented in this study are included in the article/supplementary material. Further inquiries can be directed to the corresponding author(s).

## Supplementary Materials

The following supporting information can be downloaded at https://www.mdpi.com/article/10.3390/diseases13040116/s1. Supplementary Figure S1. Flow cytometry analysis of autologous dendritic cell product.

## Abbreviations

AGEs	Advanced Glycation End-products
CKD	Chronic Kidney Disease
DC	Dendritic Cells
DKD	Diabetic Kidney Disease
DM	Diabetes Mellitus
eGFR	Estimated Glomerular Filtration Rate
ELISA	Enzyme-Linked Immunosorbent Assay
ETA	Endothelin Type A
GECs	Glomerular Endothelial Cells
GFR	Glomerular Filtration Rate
MRI	Magnetic Resonance Imaging
PI	Pulsatility Index
RAAS	Renin–Angiotensin–Aldosterone System
RI	Resistive Index
SD	Standard Deviation
SPSS	Statistical Package for Social Science
TNF-α	Tumor Necrosis Factor Alpha
UACR	Urine Albumin-to-Creatinine Ratio
VEGFR-2	Vascular Endothelial Growth Factor Receptor-2
