# Supplementary material for: The Effect of Autologous Dendritic Cell Therapy on Renal Perfusion in Diabetic Kidney Disease: Analysis of Doppler Ultrasound and Angiogenesis Biomarkers
Source: Diseases. 2025 Apr 16;13(4):116. doi: 10.3390/diseases13040116 (PMC12026179; doi:10.3390/diseases13040116)
Supplement: Supplementary file 1 [file diseases-13-00116-s001.zip › diseases-3501542-supplementary.pdf]

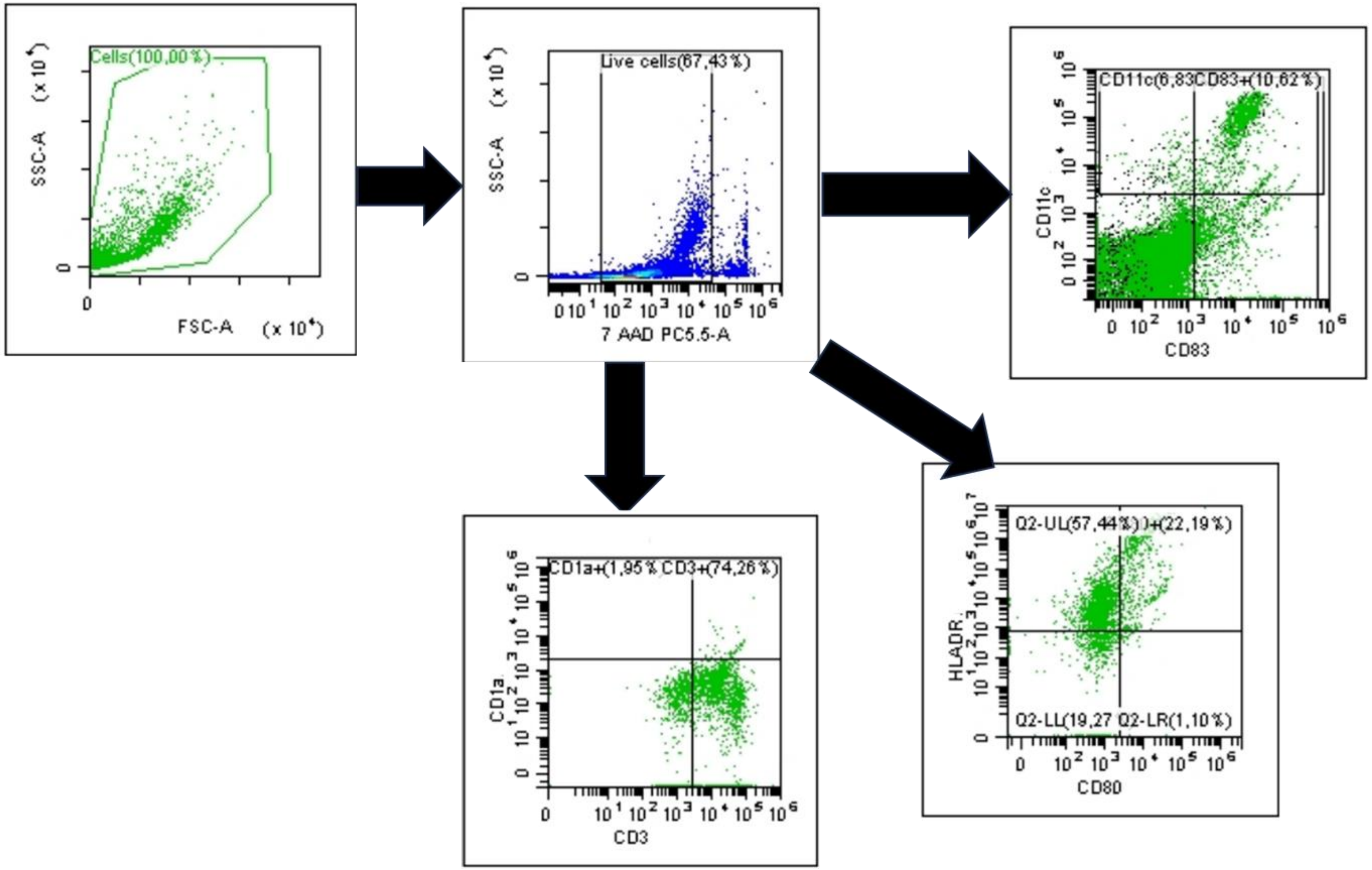

Supplementary Figure S1. Flow Cytometry Analysis of Autologous Dendritic Cell Product. Flow cytometry analysis revealed the following cellular composition of the autologous dendritic cell product: immature dendritic cells ($CD11c^+$ $CD83^-$) comprised 6.83% of the population; mature dendritic cells, as indicated $CD11^+CD83^+$, accounted for 10.62%, $HLA\text{-}DR^+CD80^-$ cells represented 57.44% and $HLA\text{-}DR^+CD80^+$ cells represented 22.19%. Langerhans-like cells ($CD1a^+$) were present at 1.95%, while lymphocytes ($CD3^+$) constituted at 74.26%.
